# Digital health and equitable access to care

**DOI:** 10.1371/journal.pdig.0000573

**Published:** 2024-09-25

**Authors:** James Shaw, Ibukun-Oluwa Omolade Abejirinde, Payal Agarwal, Simone Shahid, Danielle Martin

**Affiliations:** 1 Department of Physical Therapy, University of Toronto, Toronto, Canada; 2 Institute for Health System Solutions and Virtual Care, Women’s College Hospital, Toronto, Canada; 3 Dalla Lana School of Public Health, University of Toronto, Toronto, Canada; 4 Trillium Health Partners, Institute for Better Health, Mississaugua, Canada; 5 Department of Family and Community Medicine, Temerty of Medicine, University of Toronto, Toronto, Canada; University of Pittsburgh, UNITED STATES OF AMERICA

## Abstract

Research on digital health equity has developed in important ways especially since the onset of the COVID-19 pandemic, with a series of clear recommendations now established for policy and practice. However, research and policy addressing the health system dimensions of digital health equity is needed to examine the appropriate roles of digital technologies in enabling access to care. We use a highly cited framework by Levesque et al on patient-centered access to care and the World Health Organization’s framework on digitally enabled health systems to generate insights into the ways that digital solutions can support access to needed health care for structurally marginalized communities. Specifically, we mapped the frameworks to identify where applications of digital health do and do not support access to care, documenting which dimensions of access are under-addressed by digital health. Our analysis suggests that digital health has disproportionately focused on downstream enablers of access to care, which are low-yield when equity is the goal. We identify important opportunities for policy makers, funders and other stakeholders to attend more to digital solutions that support upstream enablement of peoples’ abilities to understand, perceive, and seek out care. These areas are an important focal point for digital interventions and have the potential to be more equity-enhancing than downstream interventions at the time that care is accessed. Overall, we highlight the importance of taking a health system perspective when considering the roles of digital technologies in enhancing or inhibiting equitable access to needed health care.

## Author summary

Our research finds that many digital health tools are focused on making it easier to talk to a health care provider or see your health care information, but that is only part of the story about how technology can help with health care. If digital technologies were used to make it easier to find health care in the first place, and easier to find information online for people who might not know the best ways to find information, these could help to make sure everyone gets the care they need.

The topic of digital health equity has attracted substantial attention in recent years, informing the development of strategies at multiple levels to enhance equitable access to digitally enabled health care [1,2]. The aim of efforts to ensure that digitally enabled health care can be accessed by all who seek to do so is certainly important. However, an exclusive focus on supporting access to common digital tools such as synchronous video visits or asynchronous patient portals might distract from dialogue on the most appropriate role(s) of digital technologies in equity-oriented health systems more generally. For example, what role might technology play in raising awareness about the broad spectrum of services available in a health system and where to find them? Or in identifying health services that others have described as culturally safe?

In this paper we examine the roles that digital health technologies can play in promoting access to health care, emphasizing the importance of a broad view of access in the effort to understand the specific opportunities presented by digital health. Using Levesque et al’s [3] patient-centered framework on access to care and the World Health Organization’s [4] (WHO) framework on uses of digital health in health systems, we generate insights into the ways that digital solutions can both support and interfere with access to health care for structurally marginalized communities. Where we use the phrase “structurally marginalized communities”, we refer to groups or communities that have experienced systemic disadvantage, discrimination, or marginalization as a result of social systems that assign privilege and oppression to groups based on characteristics such as ethnicity, income status, educational attainment, gender, sexual orientation, disability, etc [5,6].

Our analysis shows that digital health has disproportionately focused on downstream enablers of access to care, which are low-yield when equity is the goal. In response to our process of analysis, and based on recent literature and our experience promoting digital health equity in practice and policy, we propose important opportunities for policy makers, funders and other stakeholders to attend more to digital solutions that support upstream abilities to understand, perceive, and seek out needed care. In our view, these areas have the potential to be more equity-enhancing than downstream interventions at the time that care is accessed.

We identify an important distinction in the literature on digital health equity that we emphasize here at the outset of our article, which is the distinction between (a) digital determinants of health, (b) digital determinants of access to health care, and (c) digital health applications that enhance access to health care. Richardson et al [7] defined the concept of *digital determinants of health* as “conditions in the digital environment that affect a wide range of health, functioning, and quality of life outcomes and risks”. In this way, digital determinants of health are aspects of the contexts created by the increasingly pervasive nature of digital technologies that influence health and health outcomes [7]. In contrast, *digital determinants of access to health care* refers to the ways in which digital technologies serve a gatekeeping function for access to care, including for example the use of technology to engage in direct clinical care (such as a video visit), understand health needs (such as using an online symptom checker), and identify care options (such as searching online for nearby health facilities). Finally, digital health applications that enhance access to health care are those actual technology applications that serve to support a task required to access health care. In our paper we primarily address the latter two categories and remain focused on the links between digital technologies and access to health care.

Access to health care is a central determinant of whether and how health systems can equitably deliver high quality care to entire populations, and has been extensively discussed in the health systems and policy literature. Access is understood as being multi-dimensional and reaching far beyond the availability of care. It includes considerations such as whether and how patients become aware of services, geographic proximity, cultural acceptability, and appropriateness of care [8,9]. In developing a framework on health care access that is explicitly situated in the social determinants of health, Levesque et al [3] acknowledge the crucial role played by social conditions in determining capacities and barriers to accessing care. We use this widely adopted framework in this paper to explore the links specifically between access to care and the functionalities offered by digital health.

In 2019 the World Health Organization published a set of guidelines and a classification tool to guide the practical aspects of planning digital health integration to strengthen health systems. This tool represents a comprehensive framework for understanding the specific use cases for digital health as it concerns health service and planning functions. In our analysis we also draw on the WHO framework, which is a typology of health system uses of digital health, and map these uses onto Levesque et al’s framework to identify the ways in which digital health supports access to care. We find that the overlay of these two frameworks provides important insights into the links between equity, digital health, and access to care.

## The role of access in equity-oriented health systems

**Fig 1:**
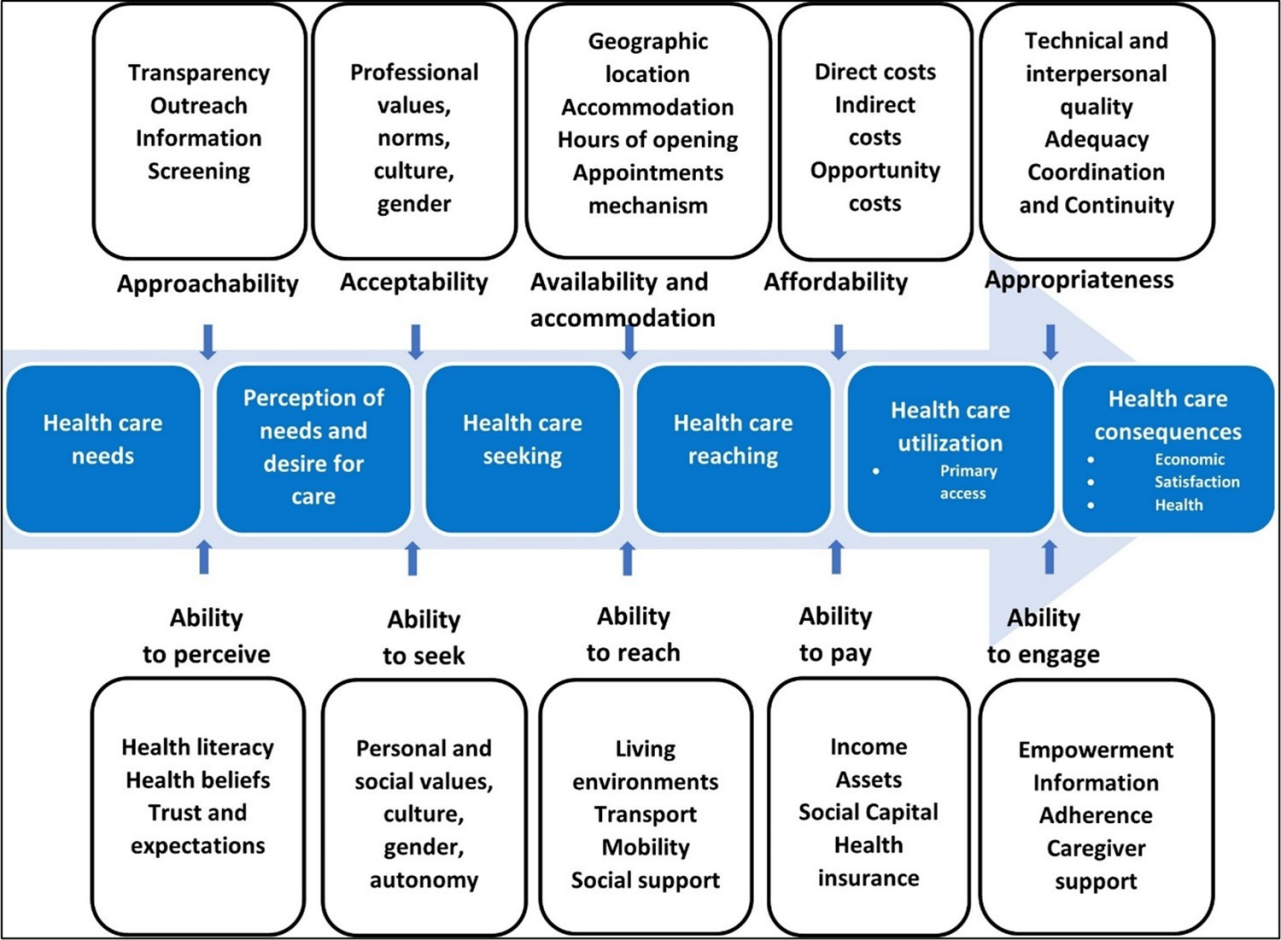
Levesque et al’s [3] Framework on Patient-Centered Access to Care.

Access to health care is a central way in which health care systems can promote or undermine equity. Where people belonging to particular communities are systematically less able to access needed care, unmet needs are experienced at a community-wide scale, leading to worse health outcomes at the sub-population level [9]. In their widely cited framework on patient-centered access to care, Levesque et al [3] defined access as the “opportunity to reach and obtain appropriate health care services in situations of perceived need for care”. Based on their definition, access is influenced by several conditions on the side of the person seeking care (the “demand side”) and the entities delivering care (the “supply side”). Levesque et al [3] produced a detailed framework outlining these conditions (reproduced in Fig 1), and pointed to future directions where action is needed to enhance access to care in health systems.

At the height of the COVID-19 pandemic, digital health and virtual care were celebrated as strategies to maintain access to health care when in-person care was discouraged. However, limited access to digital health and virtual care for some communities meant that these strategies were not equally effective for everyone in need of care, as demonstrated by a strong body of research that emerged throughout the pandemic [10–12]. Since 2020, a more sophisticated discussion has emerged on the appropriate role of digital technologies in enabling equitable access to care, exploring the instances in which digital health technologies are appropriate versus when in-person care is necessary [13,14]. Beyond the synchronous delivery of virtual care, this same line of inquiry applies to the full suite of applications of digital technologies to health care–from the use of asynchronous messaging and email to online appointment booking and many more examples. This analysis requires an understanding of the points on the journey where digital health is being taken up in health systems more broadly, which we explore specifically in this paper.

## The role of digital health in health systems

Digital health has been defined in a variety of ways. Here we treat it as a broad concept that includes the delivery of care at a distance, the digital storage and sharing of health information, and the use of health-related data to improve services and systems. This broad definition allows our analysis to include “low-tech” modalities, such as short message service (SMS), as well as “high-tech options”, such as algorithm-powered digital apps for chronic disease self-management.

Ultimately, the relevance of digital health in health systems lies in the capacity of technologies to support health systems to achieve their goals. This possibility has been much discussed in the literature [15–17], with contributions over the past several years proposing various health system pillars that can be effectively supported by digital health across country contexts [18]. Building on these discussions, the World Health Organization developed a set of guidelines and a classification tool for digital health interventions, which clearly describes the possible applications of digital health to support the objectives of health systems [4].

The WHO describes applications of digital health for health system strengthening with a specific focus on the roles of clients, health care providers, health system managers, and data services. These focal areas for applying digital health are situated in relation to health system challenges that can be addressed through digital health, organized into eight domains: (1) Information access and exchange, (2) availability of supplies and services, (3) quality of care, (4) acceptability of services, (5) utilization and demand, (6) efficiency of service delivery, (7) cost, and (8) accountability for service delivery. These eight areas of application represent the diversity of considerations necessary for planning and implementing a health system as a whole, and as such are expansive. We acknowledge that the WHO framework has been critiqued as neglecting elements that drive success in digital health, such as trust in digital technologies for health systems [19]. In this paper, our analysis is focused specifically on the applications of digital health as it affects access to health care.

## Objective

In an effort to explore how digital health can better enhance equity in access to health care, our team mapped the functions of digital health according to the WHO framework to the dimensions of access to health care as described by Levesque et al [3]. We did so to help surface those functions of digital health that support features of access to care and highlight domains in which equitable access can be enhanced through applications of digital health. We identified which features of access to care are left under-addressed by digital health as currently implemented, as a foundation for discussion about future investments in digital health from a health systems perspective.

## Methods

All authors participated in a whiteboard exercise in which the specific applications of digital health in the WHO framework were mapped to their corresponding features of access to care in the Levesque et al [3] framework. As an authorship team we have highly relevant experience for the purposes of our project, including a primary care physician with expertise in health policy leading an academic department of family and community medicine (DM), a primary care physician trained as an engineer also working as Chief Information and Innovation officer at a community hospital (PA), a research scientist trained in medicine working on the evaluation of digital health from an equity perspective (IA), a research coordinator with several years experience working on digital health projects (SS), and a social scientist trained as a physical therapist studying ethics and equity in digital health (JS). We have been studying the design, implementation, and evaluation of digital health throughout our careers, putting us in a strong position as a collaborative authorship team to complete the methods described here.

Our analysis took place through a series of four group meetings wherein we completed a visual mapping exercise, examined technologies and their applications described in the WHO framework, and refined our emerging understanding through dialogue. We approached our analysis process from a pragmatist perspective [20], drawing on the two conceptual frameworks to generate deeper insights into the conceptual and practical gaps that might exist in applications of digital technologies to enhance access to care. Our analysis was dialogic and iterative, including frequent returns to domains of the framework already addressed to check assumptions and deepen our confidence in the analysis.

We prepared a visual of the Levesque et al framework attached to a white board and used sticky notes to map applications of digital health to the features of access to care they are capable of supporting. Specifically, an application of digital health was mapped to a feature of access if it was deemed that the digital health application could facilitate that dimension of access using technology that is currently available on the market. Each feature of access to care was discussed in detail between all authors during the mapping exercise to allow sufficient opportunity for disagreement, debate, and shared understanding. For example, during the exercise, we discussed “1.3. Client-to-client communication” from the WHO framework at length, clarifying specific examples such as online patient communities and social media, and debating the ways in which such communications could enable access to needed care. We placed functions of digital health in all relevant categories of the Levesque et al framework, such that some digital health functions eventually appeared several times. To align with the supply and demand sides of the Levesque et al framework, we limited our analysis to applications of digital health pertaining to patients and health care providers.

Upon completion of the mapping exercise, we analyzed the results driven by two specific questions. The first question was, “which features of access to care are well supported by applications of digital health, and which are not?” We noted features of access to care that were over-represented and under-represented on both supply and demand sides of access, flagging these for deeper attention in our discussion. The second question was, “what do these findings suggest about the effort to leverage digital health to enable equitable access to care?” In response to the second question, we focused on areas where applications of digital health were deemed to be lacking in potential support for access to care. We structured our results section based on the most salient observations arising from our mapping exercise, and we put these observations into context in the discussion section. No ethical approval was required for this research.

## Results

**Fig 2:**
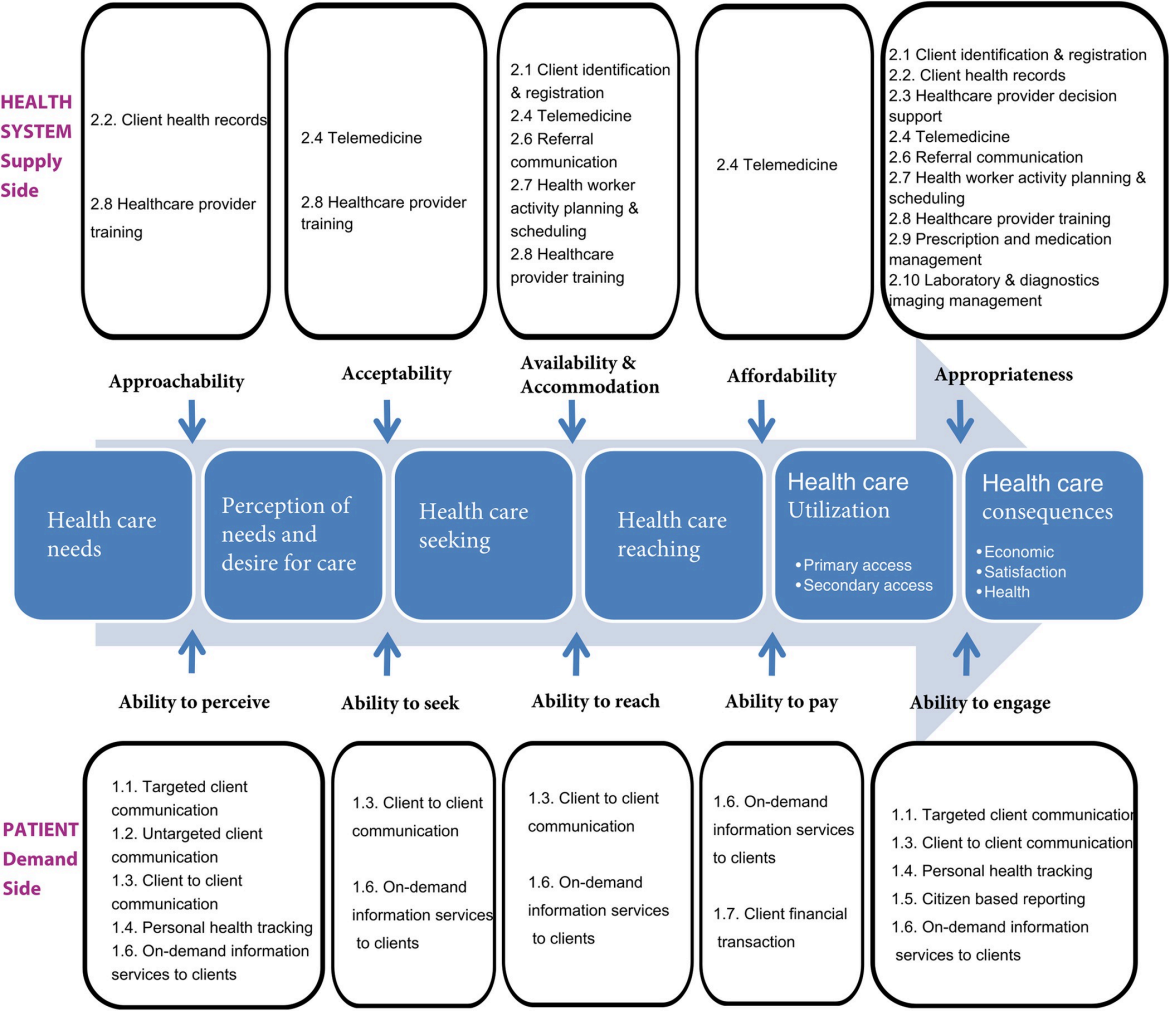
Mapping WHO categories of digital health to a framework for patient-centered access to care.

The overarching observation arising from our findings is that the majority of applications of digital health pertain to downstream features of the delivery of care, and not to the upstream features of enabling access to care on either the demand or supply side (See Table 1 for a summary). Fig 2 provides a detailed summary of our findings and visually presents the mapping of digital health applications onto the Levesque framework for access to care.

**Table 1:** Mapping WHO categories of digital health to a framework for patient-centered access to care.

Health care needs ➔ Perception of needs ➔ Care seeking ➔ Care reaching ➔ Care utilizing ➔ Health consequences					
**Health system (supply side)**	**Approachability**	**Acceptability**	**Availability and accommodation**	**Affordability**	**Appropriateness**
Number of applications	2	2	5	1	10
**Patient (demand side)**	**Ability to perceive**	**Ability to seek**	**Ability to reach**	**Ability to pay**	**Ability to engage**
Number of applications	5	2	2	2	5

### Supply side

On the supply side, we found that most digital health applications included in our analysis addressed the “appropriateness” dimension of access (10 digital applications), situated in the final stage of accessing care according to the Levesque framework. The “availability and accommodation” dimension of access, which describes the geographical (e.g., location), physical (e.g., building access), and temporal elements of access (e.g., scheduling), had the second largest number of digital health applications (5 digital applications), with two or fewer applications applying to the remaining dimensions. With respect to specific applications of digital health, “2.8. Provider training” had an outsized presence across all dimensions of access. We interpret this as a suggestion that there is high potential to incorporate training into applications of digital technology that can enhance health care providers’ abilities to provide more accessible services across the spectrum of access-related issues. The application “2.4 Telemedicine” was also present on 4 occasions on the supply side, which was unsurprising given the central role of telemedicine in providing health care services at a distance. The application “2.4 Telemedicine” was the only supply side application of digital health that could support affordability of services. These findings highlight the limited investments made thus far in digital health applications *related to the approachability*, *acceptability*, *or affordability* of services on the supply side.

### Demand side

On the demand side, we found that the “ability to engage” dimension had 5 applications of digital health, which is situated in the final stage of accessing care. The further upstream dimension of “ability to perceive” care by patients also included 5 digital health applications, with just two applications relevant to the remaining dimensions. With respect to specific digital health applications, “1.3. Client-to-client communication” and “1.6. On-demand information services to Clients” had an outsized presence across features of access. We interpret this point to suggest the central importance of acquiring information about health services from trusted sources, including information from similar peers who have experience with the services being sought. Although basic web search browsers accomplish this goal in a general sense, more specific examples of online communities of patient groups might better serve to support this aim. Furthermore, on the demand side, they point to potential for digital health to facilitate access to trusted information about services that can ultimately facilitate access to care. Applications of digital health with potential to support patients’ ability to pay included “1.6. on-demand information services to clients” and “1.7. client financial transactions”, which might facilitate capability to make payments but not affordability of services. These findings highlight the limited investments made thus far in digital health applications for patients’ abilities to *seek*, *reach*, *and pay for care* leveraging digital technologies.

## Discussion

Digital health has the potential to support health equity by facilitating access to needed care, but also risks worsening health inequities when some communities are excluded from its benefits [21,22]. When considered using a health system perspective, investments in digital health should support access for structurally marginalized communities, through the modalities that work to meet their needs and at crucial milestones along the pathway to accessing care. In our paper we have described the results of a process in which we mapped functions of digital health to a framework for access to health care, allowing us to highlight areas in need of further consideration, innovation and investment. We find that a number of access domains such as on-demand information services and patients’ ability to pay, have received comparatively less attention in digital health based on the fewer numbers of applications available to address them, highlighting areas for future work in research, policy, and technology innovation.

A strong body of literature has been developed on the ways in which digital health can be leveraged to enhance access to high quality health care for structurally marginalized communities. In a recent contribution to this field, Szymczak et al [23] situated the role of telemedicine in facilitating access to care in a context characterized by political and structural determinants of health that cause precarity and produce barriers to access for some communities. They clarify that the impacts of telemedicine in enhancing access to care are context dependent, related to the specific needs of the community of patients being offered telemedicine as a modality of care [23]. For example, where someone with low English proficiency is seeking care, what are the determinants of whether and how they come to learn about and engage with telemedicine?

We contend that understanding the impact of digital health on equitable access to care demands attention to the upstream dimensions of access that would enable that patient to engage with telemedicine later in their access journey. Upstream determinants of health care access refer to the broader social, economic, and environmental realities that influence an individual’s ability to obtain healthcare services. For example, the availability of information about local services in multiple languages, or the widespread use of a third party interpretation application that enables online information to be translated to the language of choice. Leveraging digital platforms to build and support local, culturally appropriate patient networks could provide support for patients in understanding health needs. Further upstream, giving patients full access to their health records, with a layer of digital supports to understand the information as relevant to them (their language, culture, care goals, local resources, etc.) could enable a more inclusive, equitable approach to empowering patients to understand their health needs. As outlined by Lyles et al [24], when organizations take a comprehensive strategy to build equity into their applications of digital health, issues of access will be broadly addressed across services offered.

The upstream considerations we emphasize here point toward the *digital determinants of access to health care* that either enable or constrain access to needed health services overall. Despite the importance of these upstream digital determinants of access to care, our analysis shows that digital health investments are largely tailored to address needs at the tail end of the access continuum. These issues have been taken up in recent work addressing the intersecting systems that shape whether and how people engage with technology for access to care. For example, a narrative review by Husain et al [25] explained that a lack of attention to intersectionality and digital capital has led to relatively “thin” or superficial understandings of which communities access services through digital technologies. More recently, a study by Dakin et al [26] describes the complex links between intersectional identities held by community members and the capabilities of digital health technologies, together shaping the possibilities for who can access care through digital means and how. The novel feature of our study reported here is in explicitly examining whether and how technologies might be used to address the temporally earlier and socially more complex issues linked to upstream points in the access journey (e.g., identifying culturally safe care providers or supporting ability to pay when necessary).

The WHO guidelines caution that digital health is not a panacea. The authors recognize “the innovative role that digital technologies can play in strengthening the health system,” but emphasize that “there is an equally important need to evaluate their contributing effects and ensure that such investments do not inappropriately divert resources from alternative, non-digital approaches” [4]. Digital health cannot fix inefficient or inequitable health and social policies. For example, where multiple payers function to pay health care providers for service provision, a digital health application could help to identify an appropriate funder and coordinate the transfer of funds. However, a digital health application cannot guarantee health coverage; it is ultimately the responsibility of health system policymakers to ensure universal health coverage. Our findings emphasize the point that although applications of digital health can function across the domains of access outlined in the Levesque et al [3] framework, the policy context in which digital technologies are leveraged remains foundational.

From a health system perspective, we suggest that investments in digital health should be guided by efforts to understand the entire process of recognizing the need for, and seeking out access to health care. In such an approach, the barriers to access for structurally marginalized communities are recognized and prioritized, and the upstream digital determinants of access to care are considered as important as the actual delivery of digital health services. For example, developments in public health policy are increasingly recognizing the impacts of social media on health and examining strategies to promote the health-enhancing aspects and reduce the health-detrimental aspects of social media [27]. The health effects of information search have also been widely recognized, and policy-level strategies to combat health-related misinformation are emerging [28]. Beyond these public health examples, health systems and policymakers can invest in enhancing the accessibility of information about health services through a variety of inclusive and accessible media, including online sources. Such an approach to health system planning for digital health retains a system-wide focus on access to care, prioritizing support for health equity as digital technologies become increasingly embedded in the infrastructures of health care.
